# Pictorial review of appearances post lung thermal ablation: what is normal?

**DOI:** 10.1186/1470-7330-14-S1-P7

**Published:** 2014-10-09

**Authors:** S Smith, P Jennings

**Affiliations:** 1 Ipswich Hospital, Ipswich, UK

## 

Lung thermal ablation can be used to treat both primary and secondary thoracic malignancies. Evidence to support its use, particularly with metastases from colonic primary tumours, is now strong, with survival data approaching that seen after surgery. Because of this the use of ablative techniques (particularly thermal ablation) is growing and the Royal College of Radiologists (UK) predict that the number of patients who could benefit from such treatment may reach in excess of 5,000 per year. While thermal ablation is often restricted to tertiary referral units, general radiologists are frequently expected to interpret post-treatment imaging, often performed in the context of acute complications which can occur after discharge. Although ablation is generally a safe technique with a specific mortality rate of between 1 and 2.6% and a major complication rate (as defined by the Society of Interventional Radiology as one requiring remedial action or where the patient experiences significant morbidity) of 17.1%, it is important for all radiologists to be aware of the range of post-treatment appearances. This pictorial review will cover all the normal and abnormal appearances encountered after lung ablations, based upon a review of the literature and our experience of over 200 ablations. Emphasis is given to the range of normal appearances and there will be discussion outlining when further investigation or intervention is required post ablation.

